# Patient organ and effective dose estimation in CT: comparison of four software applications

**DOI:** 10.1186/s41747-019-0130-5

**Published:** 2020-02-14

**Authors:** Cristina De Mattia, Federica Campanaro, Federica Rottoli, Paola Enrica Colombo, Andrea Pola, Angelo Vanzulli, Alberto Torresin

**Affiliations:** 1 Department of Medical Physics, ASST Grande Ospedale Metropolitano Niguarda, Piazza Ospedale Maggiore, 3, 20162 Milan, Italy; 2 Department of Energy, Politecnico di Milano, via La Masa, 34, 20156 Milan, Italy; 3 Department of Radiology, ASST Grande Ospedale Metropolitano Niguarda, Piazza Ospedale Maggiore, 3, 20162 Milan, Italy

**Keywords:** Radiation (ionizing), Radiation dosage, Radiation protection, Software, Tomography (x-ray computed)

## Abstract

**Background:**

Radiation dose in computed tomography (CT) has become a topic of high interest due to the increasing numbers of CT examinations performed worldwide. Hence, dose tracking and organ dose calculation software are increasingly used. We evaluated the organ dose variability associated with the use of different software applications or calculation methods.

**Methods:**

We tested four commercial software applications on CT protocols actually in use in our hospital: CT-Expo, NCICT, NCICTX, and Virtual Dose. We compared dose coefficients, estimated organ doses and effective doses obtained by the four software applications by varying exposure parameters. Our results were also compared with estimates reported by the software authors.

**Results:**

All four software applications showed dependence on tube voltage and volume CT dose index, while only CT-Expo was also dependent on other exposure parameters, in particular scanner model and pitch caused a variability till 50%. We found a disagreement between our results and those reported by the software authors (up to 600%), mainly due to a different extent of examined body regions. The relative range of the comparison of the four software applications was within 35% for most organs inside the scan region, but increased over the 100% for organs partially irradiated and outside the scan region. For effective doses, this variability was less evident (ranging from 9 to 36%).

**Conclusions:**

The two main sources of organ dose variability were the software application used and the scan region set. Dose estimate must be related to the process used for its calculation.

## Key points

- There is an increased interest in the risk associated with medical x-ray exposure associated with computed tomography (CT) scans.
- Several commercial software applications allow estimating organ dose for CT examinations.
- Organ dose calculation software differs on the phantom and the calculation algorithm.
- Organ dose results obtained using different software applications are not always comparable.

## Background

Radiation dose in x-ray computed tomography (CT) has become a topic of high interest due to the increasing numbers of CT examinations performed worldwide [1–5]. Studies underlined the increase over the years in the number of CT examinations resulting in an increase in the dose per capita for the population. For the USA, Brenner et al. [6, 7] reported that the number of CT examinations per year rose from 2.8 million in 1981 to 20 million in 1995 and to 62 million in 2007. In Germany, from 1996 to 2012, the annual effective dose per capita for CT examinations has more than doubled [8]. A retrospective analysis carried out in Italy (Lombardy district) between 2004 and 2014 [9] showed a 39% increase in the number of CT examinations per 1,000 residents.

A CT scan involves a dose larger than the most common radiographic procedures. Depending on the acquisition setup, the dose to the organs included in the scan region was reported to range from 15 mSv for an adult to 30 mSv for a newborn, with an average of 2–3 scans per study [7]. With the increase in the collective dose for medical exposures, there has been an increase in publications focused on radiological risk estimation [10–12].

Exposure from a diagnostic CT examination is referred to have a stochastic effect. An epidemiological study of radiation-induced tumour risk for patients undergoing CT procedures first requires an assessment of the dose delivered to the organs and tissues exposed. The organ dose is defined as the dose received by the specific organ per unit of mass. It mainly depends on patient’s anatomy, scan region, and scanner’s output. Its estimate is the basis for risk analysis. However, the dose to the organs is not an immediate information easy to be obtained. Samei et al. [13] defined its determination as a Holy Grail [13]. In the 1990s, the European Commission (Council Recommendation 1999/519/EC) encouraged the research of new methods to estimate the patient dose in CT. The general approach was the use of a Monte Carlo algorithm associated with an anthropomorphic phantom [14–16].

In this context, the first software applications for organ dose calculation were born. In general, all applications are based on the same principle: they use a set of organ doses, pre-calculated on single sections, typically 1-cm scans, which are combined to obtain the entire scan region and adjusted according to the exposure parameters in use [15, 17].

To date, several software have been introduced to calculate the organ dose in CT. Since the 1990s, there has been an evolution of calculation methods and graphic presentations, thus allowing for an easier use. When choosing a software application for organ dose CT calculation, we should consider phantoms, algorithms, reference device and validation (if available). Phantoms include the most elementary mathematical types up to hybrid voxel computational ones, which allow more reliable estimates [18]. The calculation algorithm, combined with the scanner modelling, allows to create a set of dose coefficients used to calculate the organ dose. Some software use a limited set of these coefficients and a number of correction factors to adapt the result to the reference conditions variation, such as tube voltage or phantom [19]. By reference device we mean, the scanner model used to simulate the photons histories in the Monte Carlo code. Generally, these software applications present a list of devices from which the user can select the one of interest. Obviously, older software does not include new generation scanners.

Among the software applications we analysed, the CT-Expo (G. Stamm, Hannover and H.D. Nagel, Buchholz, Germany) is the only one that uses a family of mathematical phantoms (Adam, Eva, Child, Baby), for which the body surface and organs are expressed by equations. It is also able to simulate the modulation of the beam in the CT scan and to choose between axial and spiral mode [20]. It is an application usable in the Excel® (Microsoft Corporation, Redmond, United States) environment, based on the computational method developed by Stamm and Nagel [21] for the analysis of data collected in the survey conducted in Germany in 1999 and 2002.

The National Cancer Institute CT (NCICT) dosimetry system (National Cancer Institute, Bethesda, USA) [22–24] uses hybrid voxel computational phantoms (University of Florida family). In general, voxel phantoms are defined starting from the segmentation of CT images of patients with dimension close to the reference. Non-uniform rational basis-splines surfaces are introduced in hybrid phantoms to maintain the flexibility of stylised phantoms for anatomy modifications. In this way, it is possible to adapt the stylised phantom to the reference dimension indicated by the International Commission on Radiological Protection (ICRP) for both genders [25, 26]. In addition to the adult phantoms, the software also allows to select paediatric phantoms for newborn, 1, 5, 10 or 15 years of age.

The NCICTX software (National Cancer Institute, Bethesda, USA) implements the same NCICT hybrid voxel computational phantoms family but enhanced to better adapt to the size of the patient under study, following the National Health and Nutrition Examination Survey (NHANES) IV database [27]. The NCICTX phantom library contains 100 adult males, 93 adult females, 85 males and 73 females of paediatric age, with different mass and height combinations [28], defined starting from the NCICT phantom. The NEXO[DOSE]® software (Bracco Imaging, Milan, Italy) integrates NCICTX directly in the application, without external Internet connection.

Virtual Dose, an application funded by the National Institute of Biomedical Imaging and Bioengineering (NIBIB, USA; https://www.nibib.nih.gov/), presents a ‘software as a service’ (SaaS) architecture, for which the application can be accessed remotely through a web-based interface, without the need to install the software locally [29, 30]. NEXO[DOSE]® integrates the Virtual Dose functionality through a RESTful application program interface. Its library includes a set of voxel phantoms representing men, women and children of different ages (newborn, 5, 10, and 15 years of age) [31, 32]. It also represents pregnant women, considering the three gestation trimesters, and obese patients with different mass index [33, 34].

The aim of this study was to compare these four commercial software applications (CT-Expo and NCICT as stand-alone software applications, NCICTX and Virtual Dose, integrated within the NEXO[DOSE]® radiation dose monitoring system) in terms of dosimetric data variability, both as organ dose and as effective dose, using different calculation methods, including the simulation of different exposure CT parameters.

## Methods

**Fig. 1:**
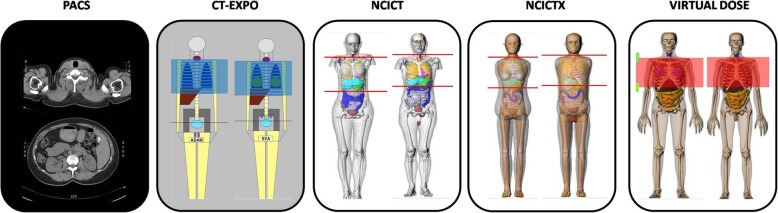
Examples of scan region on the different phantoms implemented in CT-Expo, NCICT, NCICTX, and Virtual Dose. The region to be set, in this case for a chest study, was defined by selecting images archived in our Picture Archiving and Communication System (PACS). We can see that the anatomical landmarks, used to fix the scan start and end, change according to the phantom used. In particular, CT-Expo phantom lacks anatomical details

The study was evaluated by our Institutional Review Board and the requirement for informed consent was waived. We calculated the organ doses starting from single-phase CT protocols mainly used in our hospital for head, maxillofacial, chest and abdomen-pelvis examinations. The simulated scan region was derived from a representative sample of images stored in our picture archiving and communication system while exposure parameters were extracted from the dose tracking software NEXO[Dose]®. An example of the first and last slice of a chest CT study as well as of the scan regions set on the phantom are shown in Fig. 1.

To calculate the organ dose, the software had to capture acquisition parameters such as the tube voltage (kV_p_) and the tube current (mAs). The information required by each software is reported in Table 1.

**Table 1:** Input data required by the software CT-Expo, NCICT, NCICTX, and Virtual Dose to estimate the organ dose for a CT study

Data	CT-Expo	NCICT	NCICTX	Virtual Dose
Gender (male/female)	X	X	X	X
Age	X	X	X	X
Patient habitus (weight, height)			X	X
Scan start, scan end	X	X	X	X
Scanner vendor and model	X	X	X	X
Voltage (kV_p_)	X	X	X	X
Filter (head/body)	X	X	X	X
Axial or spiral mode	X			
Current × time (mAs)	X	X	X	X
Collimation (mm)	X	X	X	X
Pitch	X	X	X	X
CTDI_vol_ (mGy)		X	X	X
Slice thickness (mm)	X			
Current modulation	X			

*CTDI*_*vol*_ Volume computed tomography dose index

For NCICTX and Virtual Dose software, where the user can select patient height and weight, we set the adult reference phantom given by ICRP 110 (2009) [35].

The four software applications do not consider the same organs for dose calculation. In addition, there are differences in organ definitions (Table 2). For example, the bone surface, one of the tissues for which the ICRP specifies the weighting factor in the effective dose calculation, has a different meaning in each of the software applications analysed. CT-Expo refers to bone surfaces, but the phantom implemented does not actually have a different bone structure for the marrow and the surface [36]. Virtual Dose considers bone endosteum instead. NCICT and NCICTX report the dose for shallow marrow tissue as opposed to the active marrow and not for the bone surface. These differences have to be taken into account in the software comparison.

**Table 2:** List of the body parts considered by CT-Expo, NCICT, NCICTX, and Virtual Dose for organ dose estimation

Body part	CT-Expo	NCICT	NCICTX	Virtual Dose
Adrenals	X	X	X	X
Bladder	X	X	X	X
Blood vessels		X	X	
Bone endosteum				X
Bone marrow	X	X	X	X
Bone surface	X			
Shallow marrow		X	X	
Brain	X	X	X	X
Breast	X (F)	X (M, F)	X (M, F)	X (M, F)
Colon		X	X	X
Effective dose ICRP 103	X		X	X
Effective dose ICRP 60	X	X	X	X
Extra-thoracic tissue	X			X
Eye balls		X	X	
Gallbladder	X	X	X	X
Gonads	X	X	X	X
Heart	X	X (wall)	X (wall)	X
Kidneys	X	X	X	X
Lens	X	X	X	
Liver	X	X	X	X
Low large intestine	X			
Lungs	X	X	X	X
Lymph nodes	X			X
Muscle	X	X	X	X
Oesophagus	X	X	X	X
Oral mucosa	X	X (oral cavity)	X (oral cavity)	X
Pancreas	X	X	X	X
Pituitary gland		X	X	
Rectum and sigmoid		X	X	
Remainder ICRP 103				X
Remainder ICRP 60				X
Salivary glands	X	X	X	X
Skin	X	X	X	X
Small intestine	X	X	X	X
Spinal cord		X	X	
Spleen	X	X	X	X
Stomach	X	X (wall)	X (wall)	X
Thymus	X	X	X	X
Thyroid	X	X	X	X
Trachea		X	X	
Upper large intestine	X			
Uterus (F)/prostate (M)	X	X	X	X

*F* Female, *ICRP* International Commission on Radiological Protection, *M* Male

We analysed the dose results at four levels. First, we estimated how the organ dose changes by varying CT exposure parameters. As reported by Hall and Brenner [10], for a CT study, the organ dose depends on a number of factors, such as the tube current and scanning time, the scan pitch, the tube voltage and the specific design of the scanner. In the organ dose calculation for single-phase protocols, we investigated the results change modifying the exposure parameters one at a time, while keeping the others fixed. Specifically, we investigated the influence of voltage, pitch, collimation and slice thickness on organ dose results. For each organ, we calculated the dose discrepancies as difference of the values obtained changing the analysed parameter. Discrepancies were then normalised to the results obtained using the parameter mainly set in the CT protocols used in our hospital and reported as percentage. For the voltage effect, we used these couples of values: 100 and 120 kV_p_, 80 and 100 kV_p_, 120 and 140 kV_p_. The values of 120, 100, and 120 kV_p_ were taken as reference, respectively. To assess the impact of slice thickness, we compared the values of 1 and 3 mm, keeping as reference the latter value, mostly used in our clinical routine. We compared the organ dose estimations resulting from setting the pitch values of 0.8, 1.0, and 1.4, the last one taken as reference. For the collimation we set 40 and 19.2 mm, keeping as reference 19.2 mm. We also investigated the influence of the scanner model. All the software analysed allowed to select vendor and scanner model. We selected the following scanner: Sensation 64 (Siemens, Syngo CT 2009E), Brilliance 64 (Philips, Host Version 3.5.5.1000), Brilliance 16 (Philips, Host Version 2.3.0.1781 ), SOMATOM Definition (Siemens, Syngo CT 2012B). For each of them, we set pitch and collimation actually in use on that device. Only for the CT-Expo software, we were able to study the organ dose discrepancies between helical and axial mode, taking the helical mode as reference.

For the other three levels of analysis, the dose results were normalised to the volume CT dose index (CTDI_vol_) used in the calculation. Thus, dimensionless values were obtained, analogous to the dose coefficients on which the software applications here considered are based [29, 37].

At the second level of analysis, NCICT and Virtual Dose results were compared with dose coefficients reported by their authors. We used as reference the organ dose coefficients reported by Lee et al. [37] and by Ding et al. [29] for head, chest and abdomen-pelvis scans, for the ICRP reference male and female adult phantoms, with 120 kV_p_. For each body part and gender, the paired Student *t* test was used. The same test was used to compare NCICT and NCICTX.

Third, we compared the organ dose results obtained from the four software applications, setting the same parameters, in particular the main used in our hospital for CT exam of head, chest, abdomen-pelvis and maxillofacial. We calculated the range (difference between maximum and minimum value) for each organ and reported it as a percent of the mean value. Organs were distinguished between those completely located inside the scan region, those only partially irradiated, those just outside the scan region and those distributed throughout the body (*e.g.*, skin, muscles), as already proposed by other authors [38]. For organs outside the scan region, only dose coefficients above 0.01 were reported.

Fourth, we calculated the effective dose, starting from organ dose results and multiplying our coefficients by the median CTDI_vol_ for each area [39]. We used the weighting factors for organs and tissues provided by the ICRP 103 (2007) [40]. We added for comparison the effective dose obtained multiplying the median dose-length product (DLP) by the conversion factors proposed by Huda et al. (*k* coefficients) [41–43].

## Results

### Variability due to exposure parameters change

At the first level of analysis, we tested the effect of changing voltage, collimation, pitch, slice thickness, and scanner model. All the four software applications showed a dependence on the tube voltage, while only CT-Expo showed a dependence on the other parameters. The relative discrepancies found for each couple of voltages are shown in Table 3. Discrepancies were calculated for each organ but we reported in the table only the minimum and the maximum discrepancies found for each class of organs. For the CT-Expo software, the discrepancies found were always of the same magnitude, less than 1%, independent of the position of the organ in relation to the scan region.

**Table 3:** Organ dose variation according to tube voltage. We compared 100 and 120 kV_p_, 80 and 100 kV_p_, and 120 and 140 kV_p_ keeping as reference respectively 120, 100, and 120 kV_p_. For each organ implemented in the four software studied, we calculated the discrepancies as difference of the values obtained for each couple of voltages. These discrepancies are then normalised to the organ dose at reference voltage. We reported here only the minimum and the maximum values of discrepancy found for each class of organs subdivided according to the position relative to the scan region

	CT-Expo (%)	NCICT (%)	NCICTX (%)	Virtual Dose (%)
100–120 kV_p_ (120 kV_p_ as reference)				
Organs inside the scan region	< 1	1–5	0–6	1–4
Partially irradiated organs	< 1	3–8	3–9	0–6
Organs outside the scan region	< 1	1–8	2–9	0–13
Distributed organs	< 1	1–9	1–9	3–6
80–100 kV_p_ (100 kV_p_ as reference)				
Organs inside the scan region	< 1	1–6	0–11	0–4
Partially irradiated organs	< 1	3–8	2–16	1–6
Organs outside the scan region	< 1	1–17	2–17	1–14
Distributed organs	< 1	2–17	2–13	3–6
120–140 kV_p_ (120 kV_p_ as reference)				
Organs inside the scan region	0	0	0	2–8
Partially irradiated organs	0	0	0	6–11
Organs outside the scan region	0	0	0	8
Distributed organs	0	0	0	3–9%

For all four software applications, the discrepancies obtained moving from 120 to 100 kV_p_ were within 10% for all the organs located inside the scan region or partially irradiated. From 100 to 80 kV_p_, the discrepancies were greater, especially for the organs outside the scan region and the distributed ones, with variations up to 17%. Using 140 kV_p_, organ dose results for CT-Expo, NCICT and NCICTX were the same obtained at 120 kV_p_. Only Virtual Dose showed variations by setting this voltage (Table 3).

Only the CT-Expo software showed a dependence on the other exposure parameters. Table 4 shows the discrepancies found. Variations were larger for organs at margins and outside the scan region. The major discrepancies were associated with the change of pitch and scanner model; as previously explained, the scanner model change involved the pitch and collimation adjustment.

**Table 4:** First level of analysis using CT-Expo: organ dose variations found according to slice thickness, pitch, collimation and scanner model. For the slice thickness, we compared 1 and 3 mm, keeping the latter as reference. We set pitch at 0.8, 1.0, and 1.4 (with 1.4 as reference), collimation at 19.2 and 40.0 mm (with 19.2 mm as reference). For each organ, we calculated the discrepancies as difference of the values obtained. These discrepancies were then normalised to the organ dose at reference condition. We reported here only the minimum and the maximum values of discrepancy found for each class of organs subdivided according to the position relative to the scan region

	Slice thickness (%)	Pitch (%)	Collimation (%)	Scanner model (%)
Organs inside the scan region	0–1	< 1	< 1	3–13
Partially irradiated organs	0–3	6–40	1–4	0–50
Organs outside the scan region	1–3	7–30	1–10	5–40
Distributed organs	0	2–6	< 2	1–2

Using the CT-Expo software, the helical mode was associated with a higher organ dose than that obtained with the axial mode, more marked for partially irradiated organs or those located outside the scan region, with a relative rise from 15 to 50%.

### Comparison with authors’ dose coefficients

Comparison between dose coefficients calculated by NCICT software and dose coefficients reported by its authors [37] revealed differences as reported in Table 5. The same comparison for dose coefficients calculated by Virtual Dose and dose coefficients reported by its authors [29] is also reported in Table 5.

**Table 5:** Second level of analysis for NCICT and Virtual Dose software: comparison with authors’ dose coefficients and between NCICT and NCICTX, for head, chest, and abdomen-pelvis scans (120 kV_p_). For each body part and gender, we reported the results of the paired Student *t* test (top) and the range of the relative discrepancies for each class of organs (bottom). In the CT abdomen-pelvis scan, the authors of Virtual Dose software do not report the dose coefficient for the organs considered as outside the scan region.

	NCICT/authors’ dose coefficient		Virtual Dose/authors’ dose coefficient		NCICT/NCICTX	
	M	F	M	F	M	F
CT head study	0.117	0.109	0.343	0.089	0.047	0.219
CT chest study	< 0.001	< 0.001	0.027	0.239	0.012	< 0.001
CT abdomen-pelvis study	0.054	0.015	0.089	0.002	0.127	0.018
CT head study						
Organs inside the scan region	1–2%	2–6%	1%	3%	13–25%	4–13%
Partially irradiated organs	24–40%	44–72%	25–29%	16–22%	4–33%	6–180%
Distributed organs	10–12%	21–30%	2–14%	10–13%	5–19%	0–21%
CT chest study						
Organs inside the scan region	2–30%	2–33%	3–9%	3–8%	3–20%	6–17%
Partially irradiated organs	19–260%	43–190%	0–4%	1–6%	20–75%	0–32%
Organs outside the scan region	21–230%	80–200%	0–16%	4–19%	0–82%	1–100%
Distributed organs	22–27%	32–34%	5–7%	4–5%	4–6%	1–5%
CT abdomen-pelvis study						
Organs inside the scan region	0–400%	0–62%	0–640%	0–22%	0–24%	0–17%
Partially irradiated organs	0%	0%	57–110%	47–83%	7–28%	10–200%
Organs outside the scan region	0-3%	2%	-	-	9–11%	19–36%
Distributed organs	19–52%	12–18%	19–33%	1–18%	14%	1–10%

*F* Female, *M* Male

The disagreement obtained is higher for the organs at the edge of the scan region and it ranges generally between 10 and 50%, but it can exceed the 100%.

### Comparison between NCICT and NCICTX

The results of the paired Student *t* test using these two software applications are shown in Table 5. They were not significantly different only for head female (*p* = 0.219) and abdomen-pelvis male areas (*p* = 0.127).

### Comparison among the four software applications

**Fig. 2:**
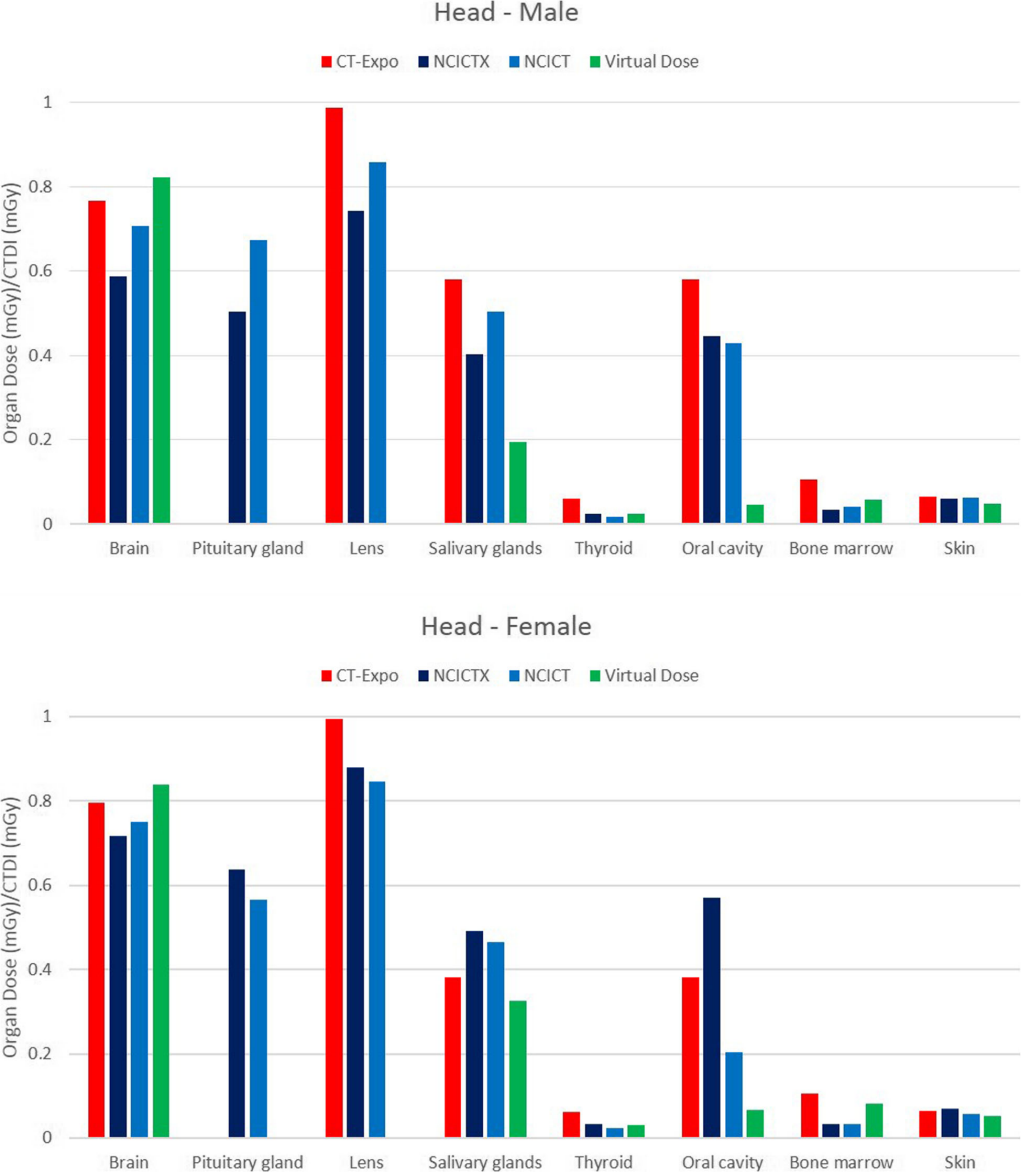
Organ dose per volume computed tomography dose index (CTDI_vol_) unit, obtained simulating the CT Head protocol with CT-Expo, NCICTX, NCICT, and Virtual Dose, using a male and a female phantom. These values are dose coefficients, obtained as organ dose (mGy) and CTDI_vol_ (mGy) ratio. Brain, pituitary gland, and lens are completely irradiated. Only NCICTX and NCICT consider the pituitary gland. Virtual Dose does not consider lens

**Fig. 3:**
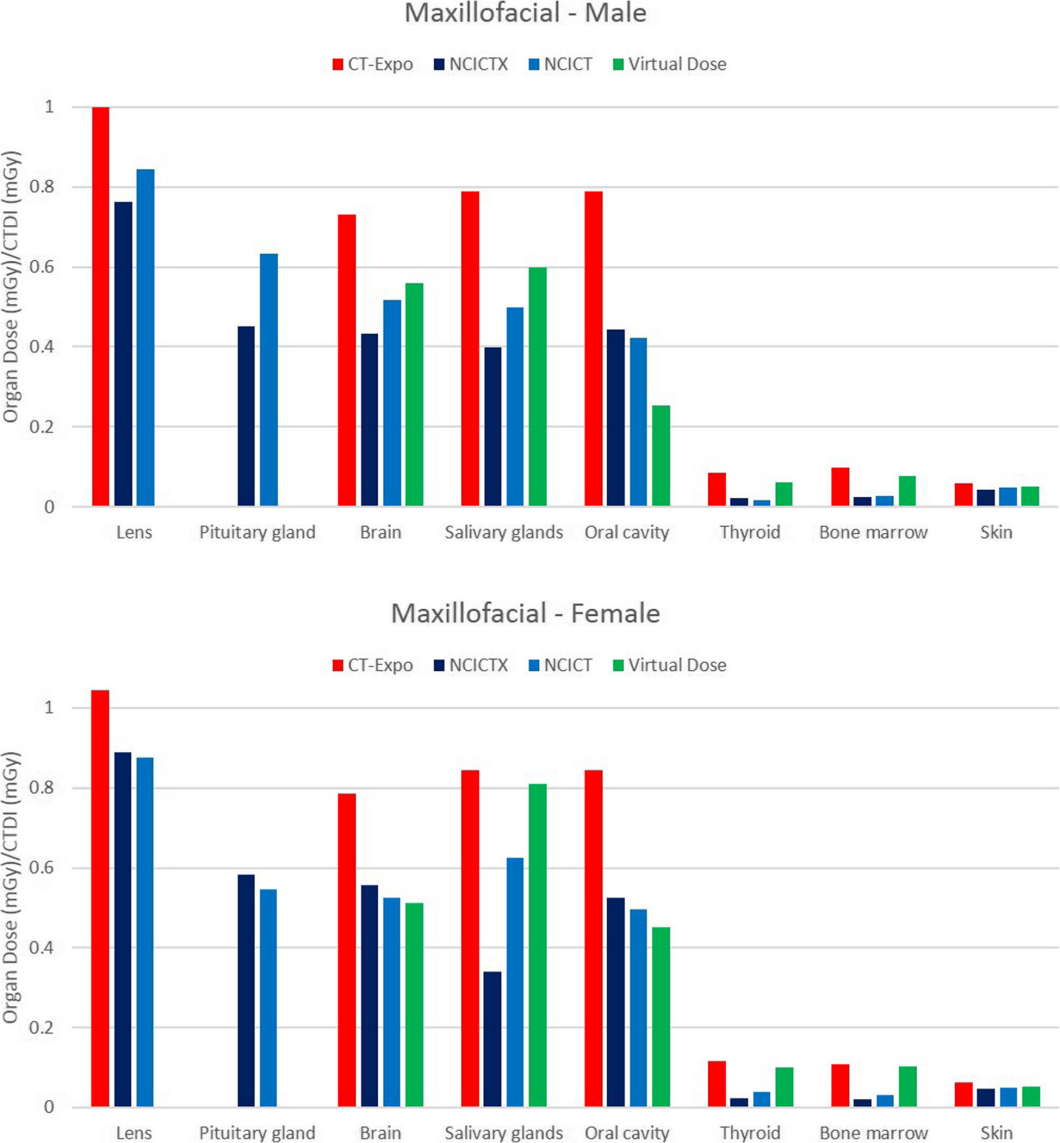
Organ dose per volume computed tomography dose index (CTDI_vol_) unit, obtained simulating the CT maxillofacial protocol with CT-Expo, NCICTX, NCICT, and Virtual Dose, using a male and a female phantom. These values are dose coefficients, obtained as organ dose (mGy) and CTDI_vol_ (mGy) ratio. Pituitary gland and lens are completely irradiated. Only NCICTX and NCICT consider the pituitary gland. Virtual Dose does not consider lens

**Fig. 4:**
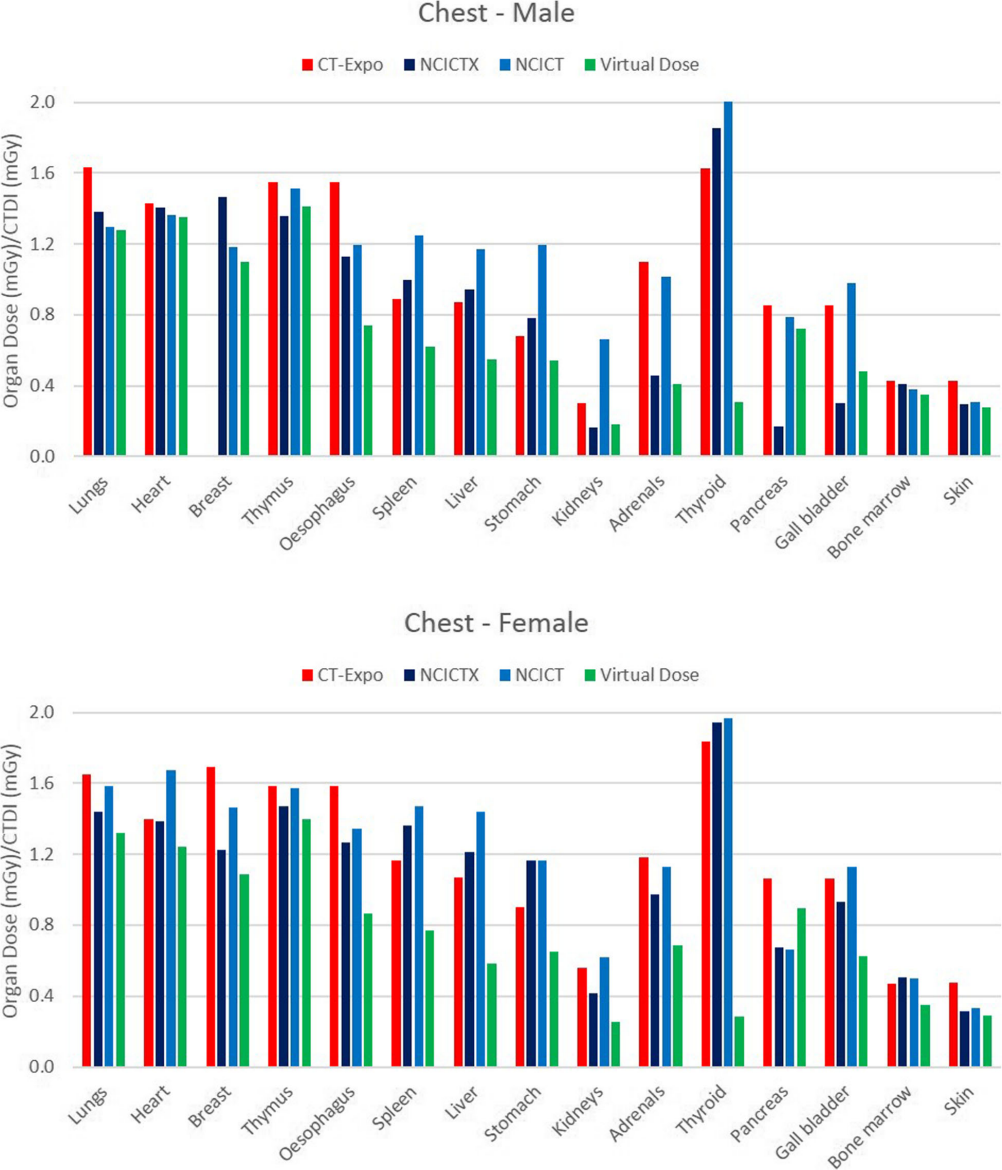
Organ dose per volume computed tomography dose index (CTDI_vol_) unit, obtained simulating the CT Chest protocol with CT-Expo, NCICTX, NCICT, and Virtual Dose, using a male and female phantom. These values are dose coefficients, obtained as organ dose (mGy) and CTDI_vol_ (mGy) ratio. Lungs, heart, breast, thymus, oesophagus, and spleen are completely irradiated. CT-Expo considers the breasts only for the female phantom

**Fig. 5:**
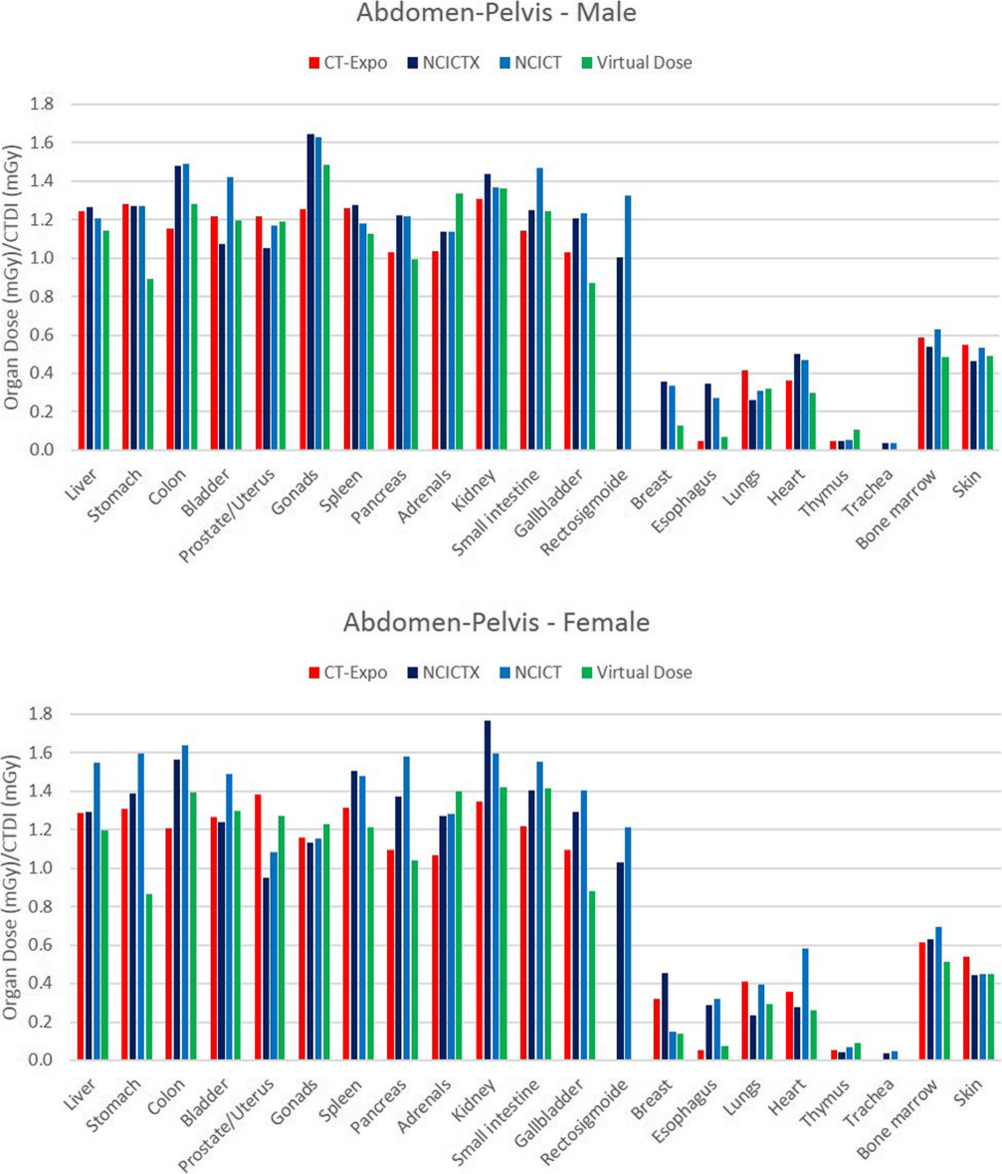
Organ dose per volume computed tomography dose index (CTDI_vol_) unit, obtained simulating the CT abdomen-pelvis protocol with CT-Expo, NCICTX, NCICT, and Virtual Dose, using a male and female phantom. These values are dose coefficients, obtained as organ dose (mGy) and CTDI_vol_ (mGy) ratio. Liver, stomach, colon, bladder, prostate/uterus, gonads, spleen, pancreas, adrenals, kidneys, small intestine, gallbladder, and rectum are completely irradiated. Only NCICTX and NCICT consider trachea and rectum. CT-Expo considers the breasts only for the female phantom

Figure 2 shows the histogram of the organ dose per CTDI_vol_ unit (dose coefficient), obtained simulating the CT head protocol with the four software applications, using male and female reference phantoms. Among the considered organs, brain, pituitary gland and lens were completely irradiated. Only NCICTX and NCICT consider the pituitary gland. Figure 3 shows the histogram of the dose coefficients obtained simulating the CT maxillofacial protocol. Figure 4 shows the histogram of the dose coefficients obtained simulating the CT chest protocol. Finally, Fig. 5 shows the histogram of the dose coefficients obtained simulating the CT abdomen-pelvis protocol.

Table 6 summarises the mean value and the relative range obtained using the four software applications according to body parts, taking into consideration the location of the organs with respect to the scan region. The range was wider for the partially irradiated organs and external to the scan region; for the organs inside the scan region, excluding oesophagus, spleen, and stomach, the range was generally within 35%.

**Table 6:** Dose coefficients obtained as an average of the values calculated by the software CT-Expo, NCICT, NCICTX, and Virtual Dose. In brackets, the range is shown as the difference between maximum and minimum, divided by the mean value

			DC (% relative range)	DC (% relative range)
			Male	Female
Organs inside the scan region	Head	Brain	0.722 (33%)	0.777 (16%)
		Pituitary gland	0.589 (29%)	0.603 (12%)
		Lens	0.863 (28%)	0.908 (16%)
	Maxillofacial	Lens	0.869 (27%)	0.909 (18%)
		Pituitary gland	0.541 (34%)	0.565 (6%)
	Chest	Lungs	1.397 (25%)	1.498 (22%)
		Heart	1.386 (6%)	1.427 (30%)
		Breast	1.25 (29%)	1.368 (44%)
		Thymus	1.458 (13%)	1.507 (12%)
		Oesophagus	1.153 (70%)	1.266 (57%)
		Spleen	0.938 (67%)	1.192 (59%)
	Abdomen-pelvis	Liver	1.215 (10%)	1.33 (27%)
		Stomach	1.18 (33%)	1.291 (56%)
		Colon	1.35 (25%)	1.451 (30%)
		Bladder	1.227 (28%)	1.323 (19%)
		Prostate/uterus	1.158 (14%)	1.173 (37%)
		Gonads	1.504 (26%)	1.169 (8%)
		Spleen	1.212 (13%)	1.378 (21%)
		Pancreas	1.115 (21%)	1.274 (42%)
		Adrenals	1.162 (26%)	1.255 (26%)
		Kidney	1.369 (10%)	1.533 (27%)
		Small intestine	1.276 (26%)	1.399 (24%)
		Gallbladder	1.085 (33%)	1.169 (45%)
		Rectosigmoide	1.165 (27%)	1.123 (16%)
Partially irradiated organs	Head	Salivary glands	0.420 (92%)	0.416 (40%)
		Thyroid	0.032 (134%)	0.038 (97%)
		Oral cavity	0.376 (142%)	0.306 (165%)
	Maxillofacial	Brain	0.56 (53%)	0.577 (46%)
		Salivary glands	0.571 (68%)	0.635 (77%)
		Oral cavity	0.477 (112%)	0.561 (68%)
	Chest	Liver	0884 (70%)	1.077 (79%)
		Stomach	0.799 (82%)	0.971 (53%)
		Kidney	0.327 (153%)	0.462 (79%)
		Adrenals	0.745 (92%)	0.983 (51%)
	Abdomen-pelvis	Breast	0.272 (85%)	0.265 (120%)
		Oesophagus	0.183 (163%)	0.184 (143%)
		Lungs	0.326 (47%)	0.334 (52%)
		Heart	0.407 (50%)	0.371 (87%)
Organs outside the scan region	Maxillofacial	Thyroid	0.046 (144%)	0.068 (131%)
	Chest	Thyroid	1.449 (117%)	1.509 (111%)
		Salivary glands	0.138 (45%)	0.149 (100%)
		Oral cavity	0.119 (78%)	0.149 (97%)
		Pancreas	0.633 (108%)	0.825 (49%)
		Colon	0.132 (255%)	0.074 (85%)
		Gallbladder	0.654 (103%)	0.938 (54%)
	Abdomen-pelvis	Thymus	0.064 (91%)	0.065 (76%)
		Trachea	0.037 (12%)	0.043 (20%)
Distributed organs	Head	Bone marrow	0.060 (121%)	0.065 (112%)
		Skin	0059 (27%)	0.061 (30%)
	Maxillofacial	Bone marrow	0.057 (130%)	0.064 (131%)
		Skin	0.05 (34%)	0.053 (32%)
	Chest	Bone marrow	0.392 (20%)	0.458 (34%)
		Skin	0.328 (46%)	0.355 (52%)
	Abdomen-pelvis	Bone marrow	0.56 (0%)	0.613 (30%)
		Skin	0.51 (17%)	0.47 (20%)

For each class of organs of Table 6, the median range of the dose coefficients and the first and third quartiles (in brackets) were as follows: organs inside the scan region, 26% (16–33%); partially irradiated organs, 80% (53–114%); organs outside the scan region, 94% (60–110%); distributed organs, 33% (25–67%).

### Effective dose comparison

**Fig. 6:**
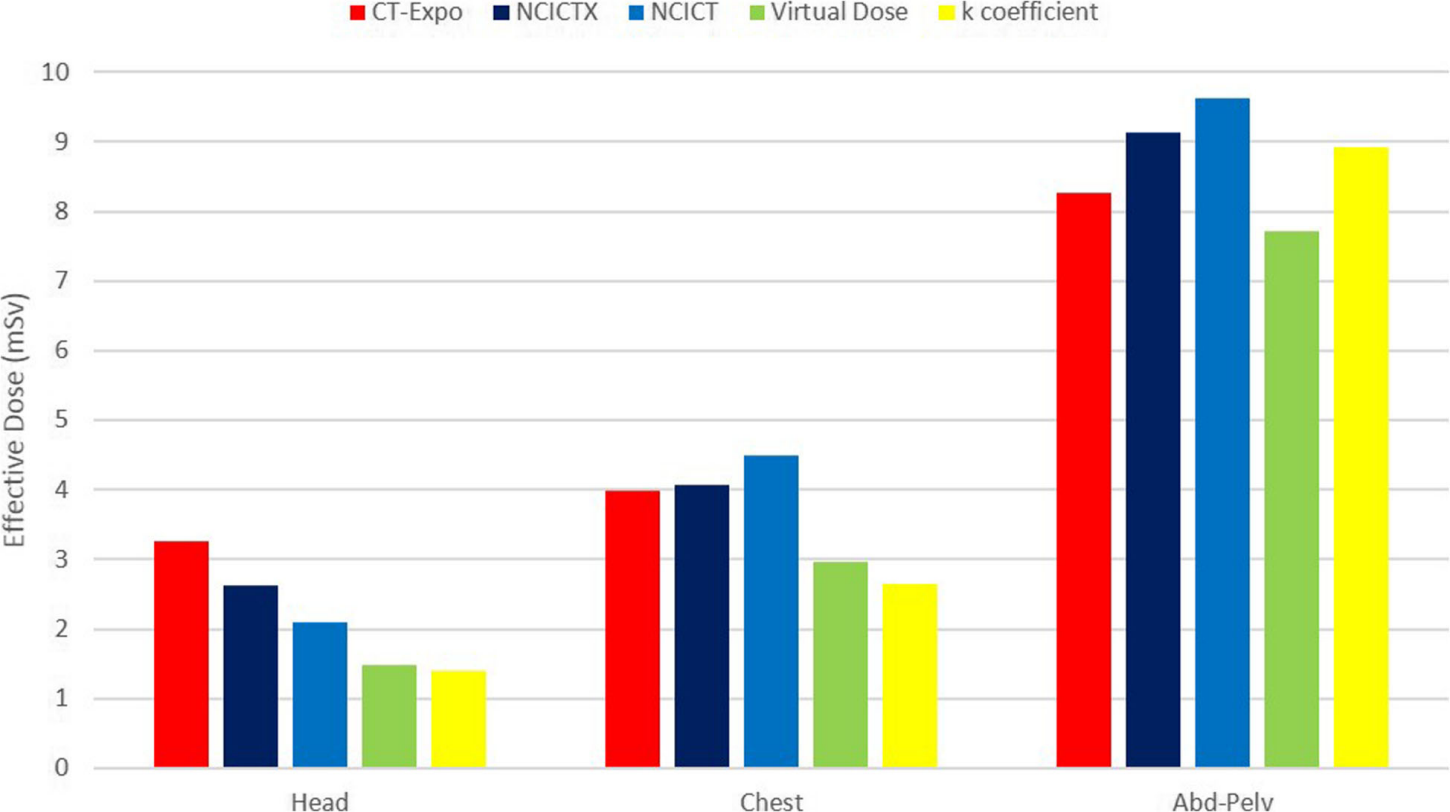
Effective dose histogram. For each software application, we estimated the effective dose starting from the organ dose coefficients multiplied by the median volume computed tomography dose index (CTDI_vol_) for head, chest, and abdomen-pelvis exams. The effective dose was obtained as sum of the organ doses weighted according to International Commission on Radiological Protection 103. We have added the values estimated using dose-length product conversion factors (*k* coefficients)

Starting from the results of the four software applications, we calculated the effective dose. In Fig. 6 we compare, through a histogram of effective doses, the software used with the value estimated using *k* coefficient multiplied by the DLP for each area. We did not reported the study of maxillofacial since there are no k coefficients for this exam. Considering all the results for each body part, the standard deviation normalised to the mean value was 36% for head, 22% for chest, and 9% for abdomen-pelvis.

## Discussion

This study showed that the variability related to the use of different software applications and methods to calculate the organ dose in CT exams is significant.

Among the software applications analysed, only NCICTX and Virtual Dose allow to adapt the phantom to the habitus of the patient, while CT-Expo is the only one able to consider the current modulation and the mode (axial/spiral), taking into account the overranging effect. Contrary to what was expected [10], only CT-Expo depends on exposure parameters such as pitch, collimation and scanner model. Though this software is more detailed in the scanner-based modelling respect to the others, at the same time, it is the least realistic from the point of view of phantom anatomy, by using stylised phantoms. It reported the same dose value for oral cavity and salivary glands, for thymus and oesophagus, for pancreas and gallbladder. For the bone marrow, CT-Expo estimated a dose much higher than those estimated by the other three software applications. This is because the phantom implemented in CT-Expo does not have a specific representation for the bone marrow and the bone surface, but it applies a correction factor to the dose received from the entire bone structure [36].

Based on our results, we can note that for all the four software applications, the only decisive exposure parameters are tube voltage and CTDI_vol_, even if we have to stress that for CT-Expo, the discrepancies found changing the tube voltage were always of the same magnitude and lower than 1%. This is probably explained by the use of conversion factors applied to a set of dose coefficients obtained at a reference voltage, while the other software applications have a set for each voltage.

In the comparison with authors’ dose coefficient, the disagreement is due basically to the different extent of the scan regions simulated by the software authors compared with those in use in our hospital. For example, in the abdomen-pelvis exam, NCICT authors specify that their scan extends from the liver up to the femoral heads, thus sparing gonads and rectum, while in our hospital, the scan extends beyond the pubic symphysis. Therefore, their values are lower than ours for bladder, rectum, prostate/uterus, and gonads, with discrepancies that exceed 100%. For Virtual Dose, gonads dose discrepancy exceeds 600%, only for male phantom, while for female, it is within 10%. These discrepancies depend on the different positions of male and female gonads in relation to the end of the scan region.

From NCICT and NCICTX applications, we expected comparable results: they were created by the same research group, with similar calculation algorithm but different phantom library. The differences found underline the importance of the phantom used. In fact, it is not easy to fix the same scan region on different phantoms, as evidenced by Fig. 1. Furthermore, the same organs can be represented with different shape, size, and position in the various phantoms, resulting in a different fractions of the irradiated organ if this is not completely included into the scan region.

The comparison among software applications showed that the variability of organ dose is lower for the completely irradiated organs than for organs partially irradiated or outside the scan region, for which the organ dose range increases. In this regard, the maxillofacial CT study is very interesting since the principal organs involved are only partially irradiated.

The range is very broad for thyroid in the chest CT study, and for salivary glands and oral mucosa in head and maxillofacial CT studies. These organs are at the border of the scan region and the scan margin definition is very critical by using different phantoms. The thyroid is always peripheral for the two mainly scanned body parts, head, and chest, but the received dose is not so negligible.

Several studies already confirmed that the dose estimation is very difficult for organs at the borders of the scan region [38, 44]. For these organs, the scattering contribution becomes important and a statistical error has to be taken into account, due to the Monte Carlo calculation uncertainties which increase while reducing the number of photons [45]. A difference of a few millimetres in the scan extent can change the dose result by some factors [36].

In terms of effective dose, variability due to the use of different software applications is less evident, except for head study for which the great percentage variation (36%) was due to the low mean effective dose. In fact, the larger dose ranges are for organs partially irradiated. Moreover, we can note that Virtual Dose returns the lowest effective dose for each scan area and that *k* coefficients seems to be too small for head and chest area.

This study has some limitations. First, we must consider the choice of the scan region. In fact, the anatomical landmarks, useful to fix the scan start and end, changed with the phantom used. This can involve a scan region of different lengths and positions on the software applications analysed. Second, using CT-Expo, the user cannot directly enter the CTDI_vol_ value. This is calculated on the basis of the mAs set. This implies that the CTDI_vol_ value used in CT-Expo may be slightly different from the other software applications. Third, a possible bias could be due to the effect of scanner model variation on dose calculation, because of our choice to change also the other parameters such as pitch or collimation, based on the aim to recreate the real-world application of the scanners in analysis. Finally, we compared the dose values in relation to the voltage set, without changing the phantom used for the calculation. However, in clinical practice, the tube voltage is linked to the constitution of the patient.

In conclusion, our study showed that (1) the organ dose value must be related to the software used and to the scan region set; (2) the dose coefficients reported in the literature for different anatomical areas represent a scan condition not always representative of the protocols used in clinical practice; (3) the acquisition parameters, such scanner model, collimation, pitch and layer thickness, do not significantly influence the dose estimation made by the software; (4) the variation in the results related to the acquisition tube voltage is lower than that due to the use of different software.

## Data Availability

The datasets used and analysed during the current study are available from the corresponding author on reasonable request.

## Abbreviations

CT: Computed tomography
CTDI_vol_: Volume CT dose index
DLP: Dose-length product
ICRP: International Commission on Radiological Protection
NCICT: National Cancer Institute CT
